# The Role of Percutaneous Ablation in the Management of Colorectal Cancer Liver Metastatic Disease

**DOI:** 10.3390/diagnostics11020308

**Published:** 2021-02-14

**Authors:** Dimitrios K. Filippiadis, Georgios Velonakis, Alexis Kelekis, Constantinos T. Sofocleous

**Affiliations:** 1 2nd Department of Radiology, Medical School, University General Hospital “ATTIKON”, National and Kapodistrian University of Athens, 12462 Athens, Greece; giorvelonakis@gmail.com (G.V.); akelekis@med.uoa.gr (A.K.); 2 Memorial Sloan Kettering Cancer Center, Weill-Cornell Medical College, New York, NY 10065, USA; sofoclec@mskcc.org

**Keywords:** ablation techniques, liver ablation, colorectal cancer, hepatic metastasis, interventional oncology, thermal ablation, microwave ablation, cryoablation

## Abstract

Approximately 50% of colorectal cancer patients will develop metastases during the course of the disease. Local or locoregional therapies for the treatment of liver metastases are used in the management of oligometastatic colorectal liver disease, especially in nonsurgical candidates. Thermal ablation (TA) is recommended in the treatment of limited liver metastases as free-standing therapy or in combination with surgery as long as all visible disease can be eradicated. Percutaneous TA has been proven as a safe and efficacious therapy offering sustained local tumor control and improved patient survival. Continuous technological advances in diagnostic imaging and guidance tools, the evolution of devices allowing for optimization of ablation parameters, as well as the ability to perform margin assessment have improved the efficacy of ablation. This allows resectable small volume diseases to be cured with percutaneous ablation. The ongoing detailed information and increasing understanding of tumor biology, genetics, and tissue biomarkers that impact oncologic outcomes as well as their implications on the results of ablation have further allowed for treatment customization and improved oncologic outcomes even in those with more aggressive tumor biology. The purpose of this review is to present the most common indications for image-guided percutaneous ablation in colorectal cancer liver metastases, to describe technical considerations, and to discuss relevant peer-reviewed evidence on this topic. The growing role of imaging and image-guidance as well as controversies regarding several devices are addressed.

## 1. Introduction

Colorectal cancer (CRC) is the second-most common neoplastic disease in Europe and a leading death cause worldwide, with a new case diagnosed every 3.5 min and a patient dying every 9 min [1,2]. Approximately 50% of CRC patients will develop liver metastases (CLM) during the course of the disease, with consequent significant increase in morbidity and mortality, and liver-only metastatic disease being present in 38% of patients dying [1,2]. Hepatic resection is a well-established local cure for CRC liver metastatic disease; however, only 10–15% CLM patients can undergo hepatectomy due to comorbidities, limited hepatic reserve, or disease extent [1,2,3,4,5]. According to the recently published European Society for Medical Oncology (ESMO) toolbox for colorectal cancer metastatic disease, local and loco-regional techniques can be used for local tumor control and improved survival rates [1]. These techniques can be applied with both curative and non-curative intents; the latter has the goal of offering the possibility of a (relevant) relapse-/disease-free interval with the minimum requirements for toxic chemotherapy [1]. According to the National Comprehensive Cancer Network (NCCN) guidelines, image-guided percutaneous thermal ablation (TA) is a local therapy indicated either alone or in conjunction with resection for CLM as long as all disease can be eradicated [6]. In oligometastatic patients, thermal ablation can be proposed as monotherapy or with hepatectomy [6] and can be combined with systemic chemotherapy, aiming to serve as a radical therapy achieving long-term local tumor control and significantly extending patient survival [3]. The combination of radiofrequency (RFA) with SOC oxaliplatin-based chemotherapy vs. chemotherapy alone has provided level I evidence regarding the effect of ablation in prolonging patient survival [3].

Currently, there are several thermal ablation (TA) technologies available, including radiofrequency (RFA), microwave (MWA), laser, or cryoablation (CWA) as well as nonthermal ablation technologies such as irreversible electroporation (IRE), otherwise known as Nanoknife. Thermal TA techniques destroy liver tumors by using different types of thermal energy, by heating (RFA, MWA, and laser), or by freezing (CWA) the target tumor; ablation with clear margins (A0) is a prerequisite for the technique in order to provide local cure and to serve as a surgical alternative. This requires proper lesion and patient selection along with high-standards application of the technique, including real-time imaging confirmation of margins. Several studies support the use of TA in the management of colorectal liver metastatic disease [3,7,8,9,10,11,12]. These techniques apply different forms of electromagnetic energy, aiming to cause irreversible cellular injury, which will lead to cellular death and tumor eradication. Evolutions in the imaging field both for diagnosis as well as for imaging guidance including F-fluorodeoxyglucose (FDG)-PET/CT, fusion, and navigation systems along with augmented reality and verification software contribute to the increased safety and efficacy rates rendering TA an attractive alternative to surgery in well-selected cases [13,14,15].

The purpose of this review is to become familiar with the most common image-guided percutaneous ablation indications for colorectal cancer liver metastatic disease, to learn about different technical considerations, and to review the current evidence. The growing role of imaging and image guidance along with controversies concerning ablation devices are discussed.

## 2. Ablation Techniques

All heat-based ablation modalities affect cell-death via the common end-points of protein denaturation and coagulative necrosis [16,17,18,19,20].

Radiofrequency ablation induces thermal damage by utilizing a high-frequency alternating current (375–500 kHz), which causes ionic oscillation, frictional heating, and coagulation necrosis as the end result [16]. Temperatures at 50 °C for 4–6 min induce cytotoxicity, whilst irreversible protein coagulation occurs between 60–100 °C [16]. Currently, in the market, there are available monopolar or bipolar radiofrequency systems with single or clustered water-cooled electrodes. Limitations and disadvantages of RFA include the presence of an active heating zone measuring a few millimeters only, along with the negative effect produced by large vessels (heat sink effect) and char residue [16]. At 100 °C, water evaporates from the tissues, causing desiccation, and the resultant electrical impedance limits the volume of thermal transmission [16,17].

Microwave ablation works similarly to RFA utilizing an electrical current produced by a 915 MHz or 2.45 GHz generator (at continuous or pulsed delivery modes) in order to cause protein denaturation and coagulative necrosis [17,18]. A water-cooled interstitial antenna is used to deliver the electrical current and to apply electromagnetic microwaves in the target lesion, creating a local non-ionizing field that interacts with dipolar molecules, causing frictional heating and coagulation necrosis as the end result. Compared to RFA, the resultant electromagnetic field is less affected by impedance, heat sink effects, low thermal conductivity, and low penetrability of the surrounding tissues [17,18]. During the past several years, MWA has gained acceptance as a favorable alternative to RFA and, in many centers, has become the preferred ablation modality. The advantages of microwave ablation include the ability to produce higher intra-tumoral temperatures, resulting in the creation of larger ablation zones in a shorter period of time; in addition, it can overcome heat sink effects and it seems to be related to lower intra-procedural pain [17,18].

Laser ablation or laser interstitial thermotherapy (LITT) utilizes a micrometer optical fiber with a bare tip that transmits infrared light (700–1200 nm) for heat production [19]. The fiber is connected to a neodymium: yttrium aluminum garnet (ND:YAG) generator or diode, which produces a precise wavelength. Factors affecting the amount the size of ablation zone include the fiber size, the wavelength used, the power and duration of the ablation session and the specific conduction and penetration parameters of the surrounding tissues [19].

Cryoablation utilizes extreme cold at temperatures of −40 °C in order to cause cellular death. One or more cryoprobes are inserted to the target lesion, aiming to create enough ice to cover the lesion and the necessary margins. Inside the cryoprobe, there is a hollow tube containing a smaller inner and a larger outer tubular part; argon gas travels from the inner to the outer tube at high pressure through a very narrow aperture, where it rapidly expands within the distal tip [20]. Due to the Joule–Thomson effect, there is heat extraction from the tip of the probe, which results in temperature drop and the formation of an ice ball [20,21]. The cryoablation session includes alternating freezing–thawing–freezing cycles. During the first freezing cycle, extracellular ice is formed, which at the thawing cycle, enters the cell due to osmotic diffusion and re-crystalizes. At the second freezing cycle, the size of intracellular crystals increases with subsequent rupture of the cellular membrane. Apart from this early cellular effect, there is a late vascular one as well, involving endothelial trauma, platelets, and microthrombi collection, with resultant ischemia and cellular death [20]. Up until now, there was limited evidence concerning the use of cryoablation for liver metastatic disease.

IRE is a non-heat-based ablation modality that utilizes electrical energy in terms of high voltage pulses that cause irreversible cellular membrane disruption and cellular death as the end result [22]. Although there is some heat development, the temperatures are sublethal at current factory-recommended settings; thus, the surrounding connective tissue should remain intact. General anesthesia and complete muscle relaxation are prerequisites for IRE in order to prevent generalized muscle contractions caused by the high voltage pulses [22]. At present, IRE for CLMs is reserved for lesions in challenging locations in an effort to prevent collateral damage mostly to central bile ducts [22].

## 3. Imaging for Diagnosis and Guidance

Pre-ablation imaging serves for patient staging and eligibility for TA. Most commonly, imaging evaluation of patients with colorectal liver metastatic disease includes a baseline computed tomography exam of the chest, abdomen, and pelvis without and with contrast (non-contrast, arterial, portal venous, and delayed phases) [6,9]. Apart from the diagnostic and staging information, the pre-contrast CT scan of the liver resembles tumor visualization on ablation day and provides information on the potential need for pre-ablation contrast medium injection, image fusion system, or any other specific guidance or navigation needs [9]. MRI with diffusion and dynamic post-contrast medium sequences is an excellent imaging technique for detecting liver lesions of small size (as small as 3–4 mm of diameter); in particular, gadoxetate disodium (gadolinium EOB DTPA, Gd-EOB-DTPA, and gadoxetic acid) is a hepato-specific paramagnetic gadolinium-based contrast agent recommended in CLM [23]. Gadolinium-EOB-enhanced MRI is the most sensitive technique to assess liver metastatic disease, and in many centers, this liver-specific MRI constitutes the technique of choice for evaluation of CLM patients. A baseline whole body FDG-PET/CT as a metabolic imaging examination provides invaluable information for disease detection and is an excellent tool for early detection of progression [12]. FDG-PET/CT is governed by proven superiority when compared to CT or MRI alone concerning the staging of CLM patients and, when added to the pre-therapeutic algorithm, could change treatment plans in patients initially considered for percutaneous ablation [24].

**Figure 1:**
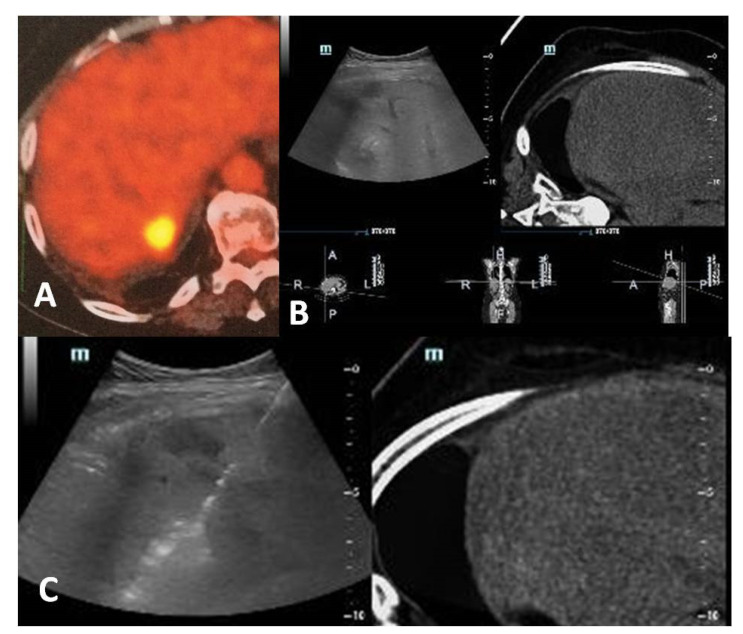
A 60-year-old, female patient with a medical record of colorectal cancer and synchronous liver metastasis for which underwent surgery and chemotherapy presented with a PET avid new hepatic lesion (**A**). Contrast-enhanced computed tomography scan was not possible due to prior severe allergic responses; fusion imaging was used for ablation guidance (**B**). A radiofrequency electrode was inserted under fusion imaging, and ablation was performed with no need for iodinated contrast medium (**C**).

**Figure 2:**
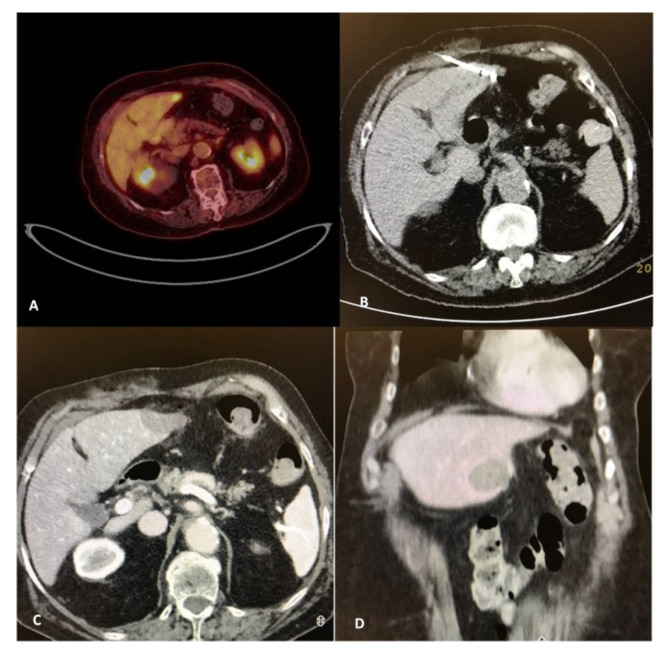
A 82-year-old, female patient with a medical record of colorectal cancer (operated) and a solitary PET avid liver metastasis depicted 9 months post-initial diagnosis (**A**). Due to comorbidities and in agreement with the patient’s desire, the tumor board decision was percutaneous microwave ablation, which was performed under computed tomography guidance (**B**). Contrast-enhanced computed tomography scan (portal venous phase) illustrates the zone of necrosis immediately post-ablation in axial (**C**) and coronal reconstructions (**D**).

**Figure 3:**
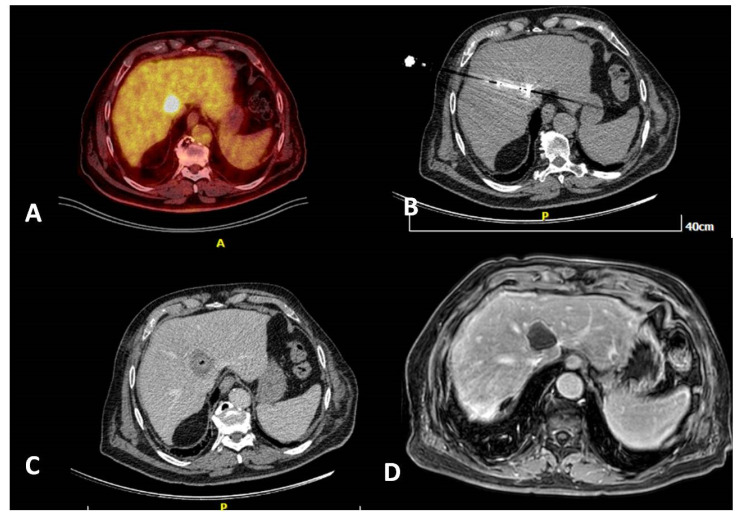
A 80-year-old, male patient with a medical record of colorectal cancer (operated) and a solitary PET avid liver metastasis right next to the junction of hepatic veins depicted 6 months post-initial diagnosis (**A**). Due to lesion location and in agreement with the patient’s will, the tumor board decision was percutaneous microwave ablation, which was performed under computed tomography guidance (**B**). Contrast-enhanced computed tomography scan (portal venous phase) illustrates the zone of necrosis immediately post-ablation (**C**). MRI 6 months post-ablation shows a lack of contrast medium uptake related to a successful ablation session (**D**).

Almost all diagnostic methods were used either solely or in combination (fusion imaging) for ablation guidance (Figure 1, Figure 2 and Figure 3). Solbiati et al. evaluated 99 patients (202 liver colorectal metastases) who underwent ultrasound-guided radiofrequency ablation combined with systemic chemotherapy, reporting 3- and 10-year survival rates equivalent to those of most surgical series reported in the literature [7]. A prior study by the same group indicated that the application of contrast enhanced ultrasound (CEUS) can decrease the rate of un-ablated lesions from 16.1 to 5.9%, associated with favorable cost-effectiveness and patients’ quality of life [25]. In a more recent study, Malone et al. concluded that the use of CEUS immediately post-ablation readily identifies residual viable tumor, enabling immediate re-treatment with resultant increases in overall treatment efficacy [26]. Mauri et al. have shown that intra-procedural CEUS seems to reduce cost per treated patient and hospital budget, allowing for immediate assessment of ablation results and reducing the number of subsequent ablations [27]. An alternative to the existing CEUS applications may be perfluorobutane contrast-enhanced ultrasonography that enables tumor visibility as discrete hypoechoic lesions [28].

Tsitskari et al. reported 96.8%, 68.7%, and 34.3% for 1-, 3-, and 5-year overall survival rates, respectively, for colorectal cancer patients with oligometastatic liver-only disease who underwent percutaneous computed tomography-guided microwave ablation [29]. Immediate assessment of margins with an intraprocedural contrast agent-enhanced CT scan and the performance of additional ablation, if necessary, has been shown to result in excellent local tumor control rates. Han et al. evaluated 365 CRC patients with 512 CLMs who underwent CT-guided RFA and immediate re-ablation in cases in which intra-procedural contrast agent-enhanced CT scan depicted tumor remnants; the authors reported 98% technical effectiveness at 92%, 41%, 30%, and 28% for 1-, 5-, 10-, and 15-year overall survival rates, respectively [30]. Shady at al. reported 94% technique effectiveness in 162 patients (233 liver colorectal metastases) that underwent computed tomography-guided ablation, whilst Ryan et al. concluded that split-dose FDG PET/CT may be a useful tool to provide both guidance and endpoint evaluation [5,31]. Cornelis et al. have shown that FDG-PET/CT, apart from guidance, can also be used as an immediate surrogate imaging biomarker of local tumor progression after percutaneous ablation of liver metastases [13]. The same authors demonstrated in a dedicated work for CLM that mean standardized uptake value (SUV) ratios were significantly higher not only in cases of viable tumor-positive immediate post-ablation biopsies but also in post-ablation negative viable tumors; thus, mean SUV ratio and minimum margin size can independently predict local tumor progression post-ablation [32]. Real-time FDG-PET/CT-guidance improves visibility, especially in less conspicuous tumors, and can improve rates of complete tumor ablation; however, it requires specific environment configurations and may increase procedure duration and cost [13]. During multi-probe stereotactic RFA, the electrodes are placed by means of an aiming device at a maximum inter-probe distance of 2 cm between the active tips throughout the tumor to the periphery of the lesion; Schullian et al. applied this technique in 64 consecutive patients with 217 recurrent CLMs post-hepatic resection, reporting 97.7% and 99.5% primary and secondary technical efficacy rates, respectively [9]. Magnetic resonance imaging for ablation guidance, however, at present is not widely available; it surpasses other imaging modalities in terms of near-real-time MR fluoroscopy for accurate applicator placement guided in different imaging planes and MR thermometry for evaluation of thermal energy delivery and lack of contrast agent need for peri-procedural assessment of the ablation zone. Winkelmann et al. evaluated 17 CRC patients with technically unresectable CLM who underwent a combination of surgical resection and MR-guided thermo-ablation, concluding that this is a safe and effective approach that can achieve long-term survival in this specific subpopulation of patients [33]. Ultrasound-based fusion imaging uses information from several prior imaging examinations into real-time guidance, combining a lack of radiation (seen in ultrasound) and an extended field of view with easy image interpretation (coming from CT or MR). The advantages of fusion imaging include increased precision and reliability, low complexity and cost, and lack of radiation exposure; however, it requires dedicated ultrasound training and registration may be complex and, at times, less accurate. Difficulties in registration are common due to differences in patient position, patient breathing, or tumor location changes, especially after intraoperative maneuvers such as hydro-dissection [14].

## 4. Clinical Applications and Patient Selection

Ruers et al. provided level I evidence that, in patients with initially unresectable liver metastatic disease, the addition of local treatment with RFA (with or without resection) to systemic FOLFOX [Folinic acid (leucovorin), Fluorouracil (5-FU), and Oxaliplatin (Eloxatin)] therapy significantly prolongs survival when compared to those treated with FOLFOX alone [3,34]. Size and number of target hepatic metastases are significant factors governing the efficacy and safety rates of percutaneous ablation; although the ideal target is a solitary lesion with a diameter <3 cm, universally acceptable indications include patients unwilling or unable to undergo surgery, with <5 metastases each measuring less than 3 cm in diameter [1,2]. According to the recent “Standards of Practice on Thermal Ablation of Liver Tumours” published by the Cardiovascular and Interventional Radiological Society of Europe, percutaneous TA techniques are indicated for up to five colorectal liver metastases, each measuring <3 cm in diameter, present in either resectable or unresectable patients [35].

Similar to the results reported by studies evaluating data from post-surgical resection of CLMs, as far as percutaneous ablation is concerned, the number of metastases (usually more than three) seems to be an independent predictor factor of tumor recurrence; Siperstein et al. evaluated 234 patients reporting a significant overall median survival difference (27 versus 17 months) associated with the presence of fewer or more than three metastases at initial presentation [36]. The size of the target lesion is a well-known predictor of local recurrence post-ablation, affecting disease-free survival; Mulier et al. evaluated 5224 hepatic tumors undergoing percutaneous and intraoperative RFA, concluding that the local recurrence rate was higher for lesions with a diameter between 3 and 5 cm when compared to lesions <3 cm in diameter [37]. Additional factors affecting overall and local tumor progression-free survival rates post-TA include lymphovascular invasion at the time of primary resection, disease-free interval from initial diagnosis to the detection of liver metastasis under 12 months, and a CEA level higher than 30 ng/mL [5,38,39,40].

Molecular and proliferation markers (Ki67, thymidylate synthase, p53, KRAS, and BRAF) may contribute to the prediction of outcomes post-ablation, offering a valuable stratification tool for tumor-tailored therapeutic approach [11]. The identification of tumor Ki67 at the end of ablation is a strong independent predictor of local tumor progression and overall patient survival, with high Ki67 ratios, suggesting that these tumors may possess an ablation resistance mechanism [11,41]. Margins over 10 mm were associated with no local progression, especially in patients with KRAS wild-type disease, whereas in RAS mutant tumors, ablation margins larger than 10 mm are mandatory to achieve local cure and sustained local tumor control [42,43,44,45,46,47].

The safety margin constitutes a significant technical factor governing local tumor control and liver progression-free survival rate; a minimum of 5 mm safety margin is a prerequisite for successful ablation, whilst margins ≥10 mm have been correlated with sustained long-term local tumor progression free survival rates >95%, as reported in the studies by Calandri et al. and Shady et al. [42,43,48]. Sotirchos et al. reported that an ablation margin of at least 5 mm accompanied by biopsy-proven complete tumor necrosis without identification of tumor cells in the ablation zone is also associated with a local progression-free survival of 97% at 30 months from treatment [41]. This may be a valuable strategy, especially in patients with underlying risk factors such as prior exposure to arterial chemotherapy or preexisting biliary dilatation, where margins over 10 mm may be an additional risk factor for biliary complication [43].

An assessment of the safety margin is of critical importance; Wang et al. evaluated 73 patients with 94 previously untreated CLM that underwent RFA, concluding that a minimal margin of the ablation zone uniformly larger than 5 mm at 4–8 weeks post-ablation CT is associated with the best local tumor control [49]. Since 2D evaluation is challenging and governed by insufficient discrimination power, multiplanar, stereotactic volumetric assessment seems to provide more reliable measurements [46,50]. Real-time assessment of the ablation zone with 3D techniques as well as the development of assays to confirm complete tumor eradication or to identify residual disease are critical for the optimization of TA [50,51,52]. Imaging software with volumetric assessment of the peri-ablational safety margin can be used for real-time ablation confirmation and are critical for the evaluation of safety margins. Trying to visually evaluate the margins around an irregular ablation volume illustrated on axial images can be biased and can end in errors. Measurement of ablation margins and thus identification of a treated patient at risk for local tumor progression can be objective and with improved discrimination value when a three-dimensional software with volumetric assessment of the peri-ablational safety margin is used intraoperatively. These software when used intraoperatively can increase not only the reliability of ablation planning but also the technique’s efficacy and safety. Laimer et al. evaluated 45 CRC patients with 76 CLM who underwent stereotactic RFA and intraoperatively used a nonrigid registration software for assessment of the percentage of predetermined peri-ablational 3D safety margins and CRLMs successfully ablated; the authors concluded that application of such a software providing image fusion of pre- and postinterventional CT scans is feasible and could represent a useful tool, indicating treatment success in daily clinical practice (100% 3D safety margin of 3 mm and at least 90% 3D safety margin of 6 mm can predict treatment success) [53].

There is currently no clear evidence supporting the superiority of one ablation technique over the other. Microwaves are less affected by the “heat-sink” effect of any kind of impedance-driven performance and achieve temperatures over 100 °C, producing a relatively larger ablation volumes in a shorter period time without the need for grounding pads placement [54,55]. Radiofrequency and microwave ablation for colorectal cancer liver metastatic disease are governed by similar technical efficacies and local progression rates, although microwaves provide better results in perivascular tumors [42].

Throughout the literature, there are limited data supporting IRE application for colorectal liver metastatic disease, focusing mainly on tumors in locations considered high risk for collateral damage if treated by TA or surgery [56]. A recent meta-analysis including all TA techniques for resectable colorectal liver metastases concluded that MWA is superior to RFA with regard to 3-year disease-free survival (60% vs. 24%) as well as overall survival (70% vs. 60%) rates [57].

## 5. Contraindications and Complications

Contraindications to TA techniques for colorectal cancer liver metastatic disease include lesions located at <1 cm distance from the main biliary duct, significant ascites along the applicator path, and untreatable or unmanageable coagulopathy [35]. 

Common side effects post-ablation of CLMs include pain and post-ablation syndrome [36]. Pain is usually not severe and self-resolving; its frequency and intensity are related to the size and of the ablation zone and its proximity to a hepatic capsule. Apart from pain 3–5 days post-ablation, a self-limited flu-like illness with low-grade fever, malaise, nausea, and/or vomiting (post-ablation syndrome) may be present, related to an inflammatory response to the ablated necrotic. Management of the aforementioned side effects is symptomatic.

Complications can be puncture- or ablation-related. Puncture-related complications include intraperitoneal bleeding, pneumothorax, and hemothorax, whilst ablation-related adverse events include bowel perforation; portal vein thrombosis; liver abscess; bile duct injury including, strictures, bilomas, and bile leaks; as well as cholecystitis [35]. Biliary injury is generally very rare, although a relatively higher risk for biliary injury has been recently reported in patients that were previously treated with intrahepatic arterial chemotherapy, preexisting biliary dilatation, and exposure to Avastin, especially when treated with margins over 10 mm [44]. Guidelines for evaluation of the bleeding risk and management of coagulopathy in patients planned for liver thermal ablation are summarized in a joint paper published by Society of Interventional Radiology(SIR), Cardiovascular and Interventional Radiological Society of Europe (CIRSE) and the Canadian Society of Interventional Radiology [58]. Complications grading from 2 to 6 according to the CIRSE classification system may appear in <5% of the treated cases [59]. Gastrointestinal perforation as a result of ablation is a rare complication (<0.2% of the cases) clearly affecting morbidity and mortality [60,61]. Ancillary techniques such as hydro- or gas dissection as well as balloon interposition techniques increase the safety of ablation whenever the target tumor lies close to a critical structure, and in the case of a peribiliary lesion, fluid instillation can be performed through the placement of a nasobiliary or biliary drainage for endoluminal cooling or warming depending on the TA technique applied [35,62].

## 6. Comparing Percutaneous Ablation to Other Therapies

Combining percutaneous TA with SOC chemotherapy for CLM is supported by the results of the EORTC-CLOCC trial that achieved long-term disease control and significant patient survival benefit (with very low morbidity) that proved the value of ablation as an adjuvant to chemotherapy over chemotherapy alone [4]. The NCCN guidelines indicate that ablation is recommended alone or in combination with resection for the treatment of CLM as long as all visible disease can be eradicated [6]. Clinical outcomes of percutaneous ablation are certainly improving, with five-year survival rates over 50% in most recent series, similar to those of surgical resection [7,8,9,10,11,12]. Comparisons of partial hepatectomy to percutaneous microwave ablation or of partial hepatectomy plus thermal ablation to larger hepatectomy resulted in comparable survival and complication rates [4]. At present, in everyday clinical practice, reasons for selecting percutaneous TA techniques over surgical approaches include a small peripheral tumor that can be ablated with margins, patients with co-morbidities, inadequate future liver remnant, patient’s choice, and difficult anatomical location of the target lesion. A non-measurable parameter affecting both treatment decision and outcome is the location of the target lesion. The extension of hepatectomy clearly associated with morbidity is related not only to lesion size but also to location. For centrally located lesions where hepatectomy would result in the removal of a large parenchymal volume, percutaneous ablation could provide similar efficacy and success at lower complication and morbidity rates without the relevant loss of liver parenchyma [63]. Percutaneous ablation can be the first choice in resectable patients too as long as the tumor can be ablated with margins and within the test of time theory that mandates close imaging follow-up; this is safer than performing resection because it allows the biology of the disease to express itself while a local cure similar to surgery is provided but with much less morbidity [64]. The test of time approach spares a significant number of patients that have multifocal progression, a morbid procedure that would offer no oncological benefit [64].

Both surgery and ablation are recommended and preferred to radiotherapy [6,15]. This is because the current evidence for stereotactic body radiotherapy (SBRT) in the management of colorectal cancer liver metastatic disease is sparse [57]. Numerous ablation papers in the literature report 3-year survival rates up to 88.6%, 5-year survival rates up to 53%, whilst longer survival rates at 7–10 years range from 18.0 to 35.9%; these rates are significantly higher than those reported after SBRT [2,29,30,31,32,33,34,46,56]. Percutaneous ablation destructs the target tumor with a safety margin of normal parenchyma sparing the uninvolved hepatic tissue. This is in opposition to SBRT application that affects a greater amount of normal liver parenchyma via the surrounding radiation portals with resultant liver toxicity [65]. Repeat treatment with SBRT is thus more likely to lead to liver toxicity or even failure. On the contrary, percutaneous ablation easily permits repeated TA with limited toxicity and affording identical survival to those that can be treated for subsequent tumors and local tumor progression (LTP) as to those treated for a solitary tumor [5,7]. Compared to SBRT, percutaneous thermal ablation offers better oncologic outcomes at a lower cost and a single session, unlike the high-cost, multiple sessions requiring SBRT [66].

## 7. Conclusions

Thermal ablation is a tumor therapy with local curative intent, offering significant and sustained local tumor control. The addition of thermal ablation to chemotherapy provides survival benefits at lower morbidity, mortality, and cost. The ablation margin is the most important, single technical factor in optimizing local tumor control. Tumor size and number, location, biology, and genetics are additional factors that can affect efficacy and safety. Percutaneous ablation can be compared only to surgical approaches in terms of survival rates offering lower complication rates. Ablation standardization in terms of predictability and reliability with the use of immediate treatment assessments is the quest of our era.
